# Peripheral blood mononuclear cells of breast cancer patients can be reprogrammed to enhance anti-HER-2/neu reactivity and overcome myeloid-derived suppressor cells

**DOI:** 10.1186/2051-1426-1-S1-P170

**Published:** 2013-11-07

**Authors:** Kyle K Payne, Christine K Zoon, Wen Wan, Khin Marlar, Rebecca C Keim, Mehrab Nasiri Kenari, Latif Kazim, Harry D Bear, Masoud H Manjili

**Affiliations:** 1 Virginia Commonwealth University - Massey Cancer Center, Richmond, VA, USA; 2 Roswell Park Cancer Institute, Buffalo, NY, USA

## 

Barriers limiting the efficacy of adoptive cellular therapy (ACT) for breast cancer patients include immune suppression mediated by myeloid-derived suppressor cells (MDSC) and a low frequency of tumor-reactive memory T cells (Tm). Recently, we developed an ex vivo protocol to reprogram tumor-reactive murine splenocytes; these cells were found to be resistant to MDSC suppression and protected FVBN202 mice from tumor challenge. Here, we evaluated the clinical applicability of reprogramming tumor-sensitized PBMCs isolated from patients with early stage breast cancer by treatment with bryostatin 1 and ionomycin (B/I) combined with IL-2, IL-7 and IL-15. Our data demonstrate that reprogrammed cells are enriched with Tm cells (n=5; p=0.006), as well as activated CD56^+^(n=6; p=0.003) and CD161^+^ (n=4; p=0.02) NKT cells, and demonstrate expansion in total cell numbers (n=16; p=0.003) compared to baseline cells. Reprogrammed PBMCs displayed enhanced HER-2/neu-specific IFN-γ producing immune responses (n=6; p=0.04); non-reprogrammed control PBMC IFN-γ production was not significant (n=6; p=0.4). Furthermore, high-throughput sequencing analysis of the T cell receptor (TcR) Vβ in one patient demonstrated clonal expansion of specific TcR VJ recombination events resulting from cellular reprogramming, suggestive of an enriched frequency of specific tumor antigen-primed T cell clones. Interestingly, reprogrammed T cells were resistant to autologous CD33^+^ CD11b^+^ HLA-DR^lo/-^ MDSCs, as determined by further enhanced HER-2/neu-specific IFN-γ secretion in the presence of MDSCs (n=6; p=0.03). Activated CD161^+^ NKT cells comprising 3% or greater of total reprogrammed cells rendered T cells resistant to MDSCs (n=3; p=0.02). Upregulation of NKG2D expression on CD161^+^ (n=5; p=0.0006) and CD56^+^ (n=5; p=0.04) NKT cells resulted from cellular reprogramming. Therefore, NKG2D signaling was blocked using anti-NKG2D blocking antibody in our co-culture system, resulting in the abrogation of resistance to MDSCs as determined by blunted IFN-γ secretion (n=3; p=0.04). Finally, the phenotype of MDSCs after co-culture with reprogrammed PBMC was examined; we observed downregulation of CD11b expression (n=3; p=0.02) concomitant with HLA-DR upregulation on MDSCs (n=3; p=0.001); suggestive of induced maturation of MDSCs into Dendritic Cells (DC). The results of our study offer the following strategies to improve ACT of breast cancer: i) inclusion of activated NKT cells in ACT to overcome MDSC suppression by inducing MDSC maturation into DCs, and ii) PBMC reprogramming to enrich the frequency of tumor-reactive Tm cells.

